# 1-(Ferrocen-1-ylmeth­yl)-3-methyl­imidazol-3-ium iodide

**DOI:** 10.1107/S1600536812045400

**Published:** 2012-11-10

**Authors:** Vincent O. Nyamori, Siphesihle M. Zulu, Bernard Omondi

**Affiliations:** a School of Chemistry and Physics, University of KwaZulu-Natal, Westville Campus, Private Bag X54001, Durban 4000, South Africa

## Abstract

The structure of the title compound, [Fe(C_5_H_5_)(C_10_H_12_N_2_)]I, consists of a 1-(ferrocen-1-ylmeth­yl)-3-methyl­imidazolium cation which is counter-balanced by an iodide anion. The cyclo­penta­dienyl (Cp) rings of the ferrocene unit have a slightly staggered conformation skewed from an ideal eclipsed conformation by an angle of 3.5 (6)°. The inter­planar angle between the Cp and the imidazole ring is 67.94 (2)°.

## Related literature

For the synthesis of ferrocenyl alkyl imidazoles, see: Simenel *et al.* (2003 ▶); Nyamori & Bala (2008 ▶). For the synthesis of ferrocenyl imidazolium salts, see: Nyamori *et al.* (2010 ▶, 2012 ▶); Bala & Coville (2007 ▶). For applications of ferrocenyl imidazolium salts, see: Gao *et al.* (2004 ▶); Ornelas (2011 ▶); Coleman *et al.* (2005 ▶); Taylor & Licence (2012 ▶).
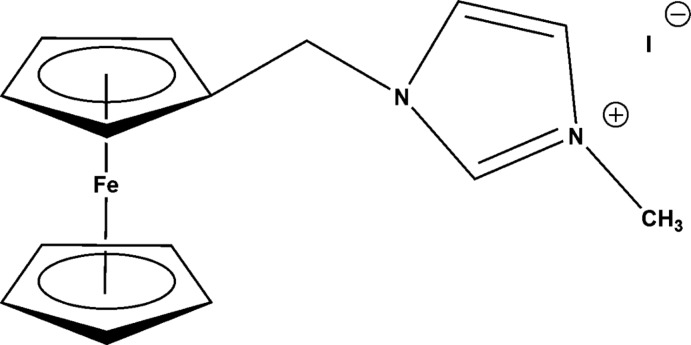


## Experimental

### 

#### Crystal data

[Fe(C_5_H_5_)(C_10_H_12_N_2_)]I
*M* *_r_* = 408.06
Monoclinic, 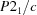

*a* = 7.2745 (3) Å
*b* = 9.3164 (3) Å
*c* = 22.2744 (9) Å
β = 90.927 (3)°
*V* = 1509.39 (10) Å^3^
*Z* = 4
Cu *K*α radiation
μ = 23.96 mm^−1^
*T* = 173 K
0.16 × 0.12 × 0.07 mm

#### Data collection

Agilent SuperNova (Dual, Cu at zero, Atlas) diffractometer
Absorption correction: analytical [*CrysAlis PRO* (Agilent, 2012 ▶), based on expressions of Clark & Reid (1995 ▶)] *T*_min_ = 0.114, *T*_max_ = 0.285
6978 measured reflections
2972 independent reflections
2745 reflections with *I* > 2σ(*I*)
*R*_int_ = 0.042

#### Refinement

*R*[*F* ^2^ > 2σ(*F* ^2^)] = 0.049
*wR*(*F* ^2^) = 0.134
*S* = 1.20
2972 reflections
173 parameters
H-atom parameters constrained
Δρ_max_ = 2.51 e Å^−3^
Δρ_min_ = −1.53 e Å^−3^

### 

Data collection: *CrysAlis PRO* (Agilent, 2012 ▶); cell refinement: *CrysAlis PRO*; data reduction: *CrysAlis PRO*; program(s) used to solve structure: *SHELXS97* (Sheldrick, 2008 ▶); program(s) used to refine structure: *SHELXL97* (Sheldrick, 2008 ▶); molecular graphics: *ORTEP-3* (Farrugia, 2012 ▶); software used to prepare material for publication: *WinGX* (Farrugia, 2012 ▶).

## Supplementary Material

Crystal structure: contains datablock(s) global, I. DOI: 10.1107/S1600536812045400/hg5258sup1.cif

Structure factors: contains datablock(s) I. DOI: 10.1107/S1600536812045400/hg5258Isup2.hkl

Additional supplementary materials:  crystallographic information; 3D view; checkCIF report

## Figures and Tables

**Fig. 1.**
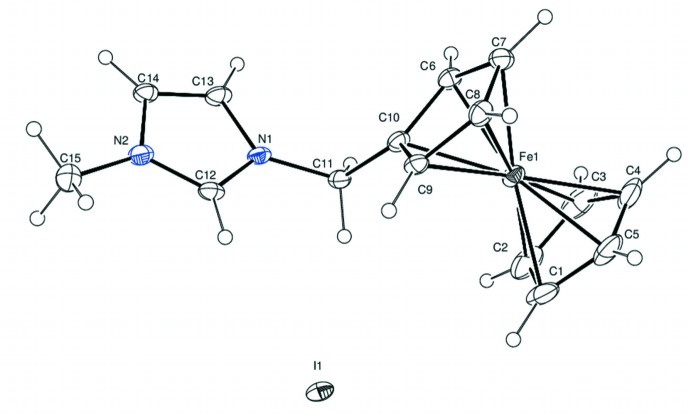
The asymmetric unit of the title compound with displacement ellipsoids drawn at the 50% probability level.

## supplementary crystallographic information

### Comment

The versatility of ferrocene has seen it be derivatized into a variety of compounds. Because of its stability and unique electronic and redox properties, ferrocene and its derivatives have been successfully applied with much success in various fields including medicinal chemistry (Ornelas, 2011, Simenel *et al.*, 2003). Ferrocenyl imidazolium salts are a special class of ferrocene derivatives which have a cationic ferrocene-imidazole moiety [FcIm^+^] balanced by an inorganic anion *X*^-^. These compounds can be applied as ionic liquids (Taylor & Licence, 2012; Gao *et al.*, 2004) and as precursors to ferrocenated imidazolium carbenes (Coleman *et al.*, 2005). The title compound was synthesized *via* solvent-free conditions providing an economical and clean product requiring less tidious purification process similar to reactions by Bala and Coville (2007) in which one of the reagents is a liquid and acts as a solvating medium.

The average bond lengths between the iron atom (Fe1) and the centroids of the substituted Cp ring (C6–C10) and the unsubstituted Cp ring (C1–C5) are 2.0382 (6) and 2.0354(7.4). The cyclopentadienyl rings of the ferrocene moiety have a slightly staggered conformation. The staggering angle between the two rings is 3.5 (6)° which is smaller than that of Nyamori & Bala (2008), Nyamori *et al.*, (2010) and Nyamori *et al.*, (2012)

### Experimental

In a round bottom flask, methyl iodide (1.64 ml, 3.75 g, 26.4 mmol) was added to 1-Ferrocenylmethyl-1*H*-imidazole (2.00 g, 7.50 mmol) and allowed to reflux for 12 hrs, at 50 °C under innert atmosphere. The reaction mixture was then allowed to cool to room temperature and washed with anhydrous diethylether (3 *x* 10 ml). A yellow solid was obtained, which was further dried *invacuo*. Yellow crystals crystals were obtained from recrystallization in diethylether (2.78 g, 91%); m.p. 134–136 °C (lit. 130–135 °C); IR (ATR cm-1) 3430, 3042, 1567, 1549, 1429, 1150, 1040, 837, 820, 755, 620, 500, 482; ^1^H NMR (CDCl_3_) 10.13 (1*H*, s, NCH), 7.16 (2*H*, m, NCH), 5.38 (1*H*, q, CH), 4.43 (2*H*, t, C_5_H_4_), 4.32 (2*H*, t, C_5_H_4_), 4.29 (5*H*, s, C_5_H_5_), 4.06 (3*H*, s, NCH_3_); ^13^C NMR (CDCl_3_) 136.12, 123.63, 121.91, 78.83, 70.09, 69.46, 50.12, 37.15; m/*z* (ESI) [*M*+] - I 281. (100%), 199 (60%); Anal. Calc. for C_15_H_17_N_2_Fe^+^ 281.07411.

### Refinement

Carbon-bound H-atoms were placed in calculated positions [C—H = 0.98 Å for Me H atoms, 0.99 Å for Methylene H atoms, 0.99–1.00 for methine H atoms and 0.95 Å for aromatic H atoms; *U*_iso_(H) = 1.2*U*_eq_(C) (1.5 for Me groups)] and were included in the refinement in the riding model approximation.

### Crystal data

**Table:**

[Fe(C_5_H_5_)(C_10_H_12_N_2_)]I	*F*(000) = 800
*M**_r_* = 408.06	*D*_x_ = 1.796 Mg m^−^^3^
Monoclinic, *P*2_1_/*c*	Cu *K*α radiation, λ = 1.54184 Å
Hall symbol: -P 2ybc	Cell parameters from 7295 reflections
*a* = 7.2745 (3) Å	θ = 4.0–74°
*b* = 9.3164 (3) Å	µ = 23.96 mm^−^^1^
*c* = 22.2744 (9) Å	*T* = 173 K
β = 90.927 (3)°	Block, yellow
*V* = 1509.39 (10) Å^3^	0.16 × 0.12 × 0.07 mm
*Z* = 4	

### Data collection

**Table:**

Agilent SuperNova (Dual, Cu at zero, Atlas) diffractometer	2745 reflections with *I* > 2σ(*I*)
Mirror monochromator	*R*_int_ = 0.042
ω scans	θ_max_ = 74°, θ_min_ = 4.0°
Absorption correction: analytical [*CrysAlis PRO* (Agilent, 2012), based on expressions of Clark & Reid (1995)]	*h* = −6→8
*T*_min_ = 0.114, *T*_max_ = 0.285	*k* = −10→11
6978 measured reflections	*l* = −19→27
2972 independent reflections	

### Refinement

**Table:**

Refinement on *F*^2^	Primary atom site location: structure-invariant direct methods
Least-squares matrix: full	Secondary atom site location: difference Fourier map
*R*[*F*^2^ > 2σ(*F*^2^)] = 0.049	Hydrogen site location: inferred from neighbouring sites
*wR*(*F*^2^) = 0.134	H-atom parameters constrained
*S* = 1.20	*w* = 1/[σ^2^(*F*_o_^2^) + (0.0682*P*)^2^ + 3.7636*P*] where *P* = (*F*_o_^2^ + 2*F*_c_^2^)/3
2972 reflections	(Δ/σ)_max_ = 0.011
173 parameters	Δρ_max_ = 2.51 e Å^−^^3^
0 restraints	Δρ_min_ = −1.53 e Å^−^^3^

### Special details

**Table:**

Geometry. All e.s.d.'s (except the e.s.d. in the dihedral angle between two l.s. planes) are estimated using the full covariance matrix. The cell e.s.d.'s are taken into account individually in the estimation of e.s.d.'s in distances, angles and torsion angles; correlations between e.s.d.'s in cell parameters are only used when they are defined by crystal symmetry. An approximate (isotropic) treatment of cell e.s.d.'s is used for estimating e.s.d.'s involving l.s. planes.
Refinement. Refinement of *F*^2^ against ALL reflections. The weighted *R*-factor *wR* and goodness of fit *S* are based on *F*^2^, conventional *R*-factors *R* are based on *F*, with *F* set to zero for negative *F*^2^. The threshold expression of *F*^2^ > σ(*F*^2^) is used only for calculating *R*-factors(gt) *etc*. and is not relevant to the choice of reflections for refinement. *R*-factors based on *F*^2^ are statistically about twice as large as those based on *F*, and *R*- factors based on ALL data will be even larger.

### Fractional atomic coordinates and isotropic or equivalent isotropic displacement parameters (Å^2^)

**Table:**

	*x*	*y*	*z*	*U*_iso_*/*U*_eq_	
C1	0.3124 (13)	−0.0474 (8)	0.7098 (3)	0.0455 (18)	
H1	0.3738	−0.1201	0.6839	0.055*	
C2	0.4011 (13)	0.0604 (11)	0.7444 (4)	0.056 (2)	
H2	0.5369	0.0768	0.7472	0.067*	
C3	0.2664 (16)	0.1406 (10)	0.7745 (4)	0.059 (3)	
H3	0.2886	0.223	0.8025	0.071*	
C4	0.0912 (15)	0.0802 (9)	0.7577 (4)	0.058 (2)	
H4	−0.0314	0.1148	0.7714	0.07*	
C5	0.1209 (13)	−0.0329 (8)	0.7183 (4)	0.0477 (19)	
H5	0.0231	−0.0932	0.6988	0.057*	
C6	0.2546 (8)	0.3596 (6)	0.6585 (3)	0.0260 (12)	
H6	0.2877	0.4416	0.6855	0.031*	
C7	0.0720 (8)	0.3107 (7)	0.6449 (3)	0.0284 (12)	
H7	−0.0445	0.3519	0.6608	0.034*	
C8	0.0869 (8)	0.1903 (7)	0.6049 (3)	0.0276 (12)	
H8	−0.0176	0.1321	0.5883	0.033*	
C9	0.2748 (8)	0.1664 (6)	0.5945 (3)	0.0244 (11)	
H9	0.3262	0.0884	0.5689	0.029*	
C10	0.3810 (8)	0.2713 (6)	0.6275 (3)	0.0243 (11)	
C11	0.5858 (8)	0.2808 (6)	0.6288 (3)	0.0254 (12)	
H11A	0.6262	0.342	0.6629	0.031*	
H11B	0.6384	0.1839	0.635	0.031*	
C12	0.6907 (8)	0.2666 (6)	0.5229 (3)	0.0258 (11)	
H12	0.6752	0.1659	0.5186	0.031*	
C13	0.6956 (8)	0.4835 (7)	0.5609 (3)	0.0294 (13)	
H13	0.6849	0.5608	0.5885	0.035*	
C14	0.7537 (8)	0.4932 (7)	0.5038 (3)	0.0278 (12)	
H14	0.7892	0.578	0.4833	0.033*	
C15	0.8032 (9)	0.3090 (9)	0.4206 (3)	0.0357 (15)	
H15A	0.9338	0.2831	0.4207	0.053*	
H15B	0.7813	0.3881	0.3924	0.053*	
H15C	0.7293	0.2257	0.4083	0.053*	
N1	0.6545 (7)	0.3416 (5)	0.5722 (2)	0.0233 (10)	
N2	0.7512 (7)	0.3536 (6)	0.4809 (2)	0.0274 (10)	
Fe1	0.22750 (13)	0.15195 (10)	0.68399 (4)	0.0244 (2)	
I1	0.69574 (5)	−0.11859 (4)	0.579436 (17)	0.02780 (16)	

### Atomic displacement parameters (Å^2^)

**Table:**

	*U*^11^	*U*^22^	*U*^33^	*U*^12^	*U*^13^	*U*^23^
C1	0.080 (6)	0.023 (3)	0.034 (3)	0.010 (3)	0.005 (3)	0.010 (3)
C2	0.060 (5)	0.059 (6)	0.047 (4)	−0.006 (4)	−0.016 (4)	0.032 (4)
C3	0.115 (8)	0.036 (4)	0.026 (3)	−0.014 (5)	−0.005 (4)	0.003 (3)
C4	0.086 (6)	0.035 (4)	0.054 (5)	−0.004 (4)	0.037 (5)	0.016 (4)
C5	0.071 (5)	0.023 (3)	0.049 (4)	−0.016 (3)	0.009 (4)	0.008 (3)
C6	0.030 (3)	0.017 (3)	0.031 (3)	−0.004 (2)	0.000 (2)	0.000 (2)
C7	0.025 (3)	0.022 (3)	0.037 (3)	0.001 (2)	0.000 (2)	0.001 (2)
C8	0.027 (3)	0.025 (3)	0.031 (3)	−0.007 (2)	−0.001 (2)	0.002 (2)
C9	0.030 (3)	0.015 (3)	0.028 (3)	0.000 (2)	0.000 (2)	0.000 (2)
C10	0.026 (3)	0.017 (3)	0.030 (3)	−0.003 (2)	0.000 (2)	0.003 (2)
C11	0.023 (3)	0.021 (3)	0.032 (3)	0.004 (2)	0.000 (2)	0.005 (2)
C12	0.024 (3)	0.016 (3)	0.037 (3)	0.003 (2)	−0.001 (2)	−0.002 (2)
C13	0.029 (3)	0.014 (3)	0.045 (4)	−0.002 (2)	−0.001 (2)	0.001 (2)
C14	0.023 (3)	0.016 (3)	0.045 (3)	−0.001 (2)	0.003 (2)	0.003 (2)
C15	0.041 (4)	0.035 (4)	0.032 (3)	0.001 (3)	0.006 (3)	0.002 (3)
N1	0.023 (2)	0.014 (2)	0.033 (3)	0.0008 (18)	−0.0016 (18)	0.0024 (19)
N2	0.026 (2)	0.021 (2)	0.035 (3)	0.002 (2)	−0.0005 (19)	0.004 (2)
Fe1	0.0326 (5)	0.0163 (4)	0.0243 (4)	−0.0031 (4)	0.0020 (3)	−0.0002 (3)
I1	0.0357 (2)	0.0139 (2)	0.0336 (2)	0.00001 (13)	−0.00212 (15)	0.00010 (12)

### Geometric parameters (Å, º)

**Table:**

C1—C2	1.416 (13)	C8—Fe1	2.054 (6)
C1—C5	1.415 (13)	C8—H8	1
C1—Fe1	2.036 (7)	C9—C10	1.441 (8)
C1—H1	1	C9—Fe1	2.033 (6)
C2—C3	1.410 (15)	C9—H9	1
C2—Fe1	2.020 (8)	C10—C11	1.492 (8)
C2—H2	1	C10—Fe1	2.028 (6)
C3—C4	1.438 (15)	C11—N1	1.476 (7)
C3—Fe1	2.034 (8)	C11—H11A	0.99
C3—H3	1	C11—H11B	0.99
C4—C5	1.390 (12)	C12—N2	1.319 (8)
C4—Fe1	2.044 (7)	C12—N1	1.333 (8)
C4—H4	1	C12—H12	0.95
C5—Fe1	2.042 (7)	C13—C14	1.350 (9)
C5—H5	1	C13—N1	1.380 (7)
C6—C10	1.420 (8)	C13—H13	0.95
C6—C7	1.432 (8)	C14—N2	1.397 (8)
C6—Fe1	2.027 (6)	C14—H14	0.95
C6—H6	1	C15—N2	1.461 (8)
C7—C8	1.437 (9)	C15—H15A	0.98
C7—Fe1	2.048 (6)	C15—H15B	0.98
C7—H7	1	C15—H15C	0.98
C8—C9	1.408 (8)		
			
C2—C1—C5	107.5 (7)	N2—C12—N1	109.6 (5)
C2—C1—Fe1	68.9 (4)	N2—C12—H12	125.2
C5—C1—Fe1	69.9 (4)	N1—C12—H12	125.2
C2—C1—H1	126.3	C14—C13—N1	108.0 (6)
C5—C1—H1	126.3	C14—C13—H13	126
Fe1—C1—H1	126.3	N1—C13—H13	126
C3—C2—C1	108.7 (8)	C13—C14—N2	106.2 (5)
C3—C2—Fe1	70.2 (5)	C13—C14—H14	126.9
C1—C2—Fe1	70.2 (4)	N2—C14—H14	126.9
C3—C2—H2	125.6	N2—C15—H15A	109.5
C1—C2—H2	125.6	N2—C15—H15B	109.5
Fe1—C2—H2	125.6	H15A—C15—H15B	109.5
C2—C3—C4	106.8 (8)	N2—C15—H15C	109.5
C2—C3—Fe1	69.1 (4)	H15A—C15—H15C	109.5
C4—C3—Fe1	69.7 (5)	H15B—C15—H15C	109.5
C2—C3—H3	126.6	C12—N1—C13	107.8 (5)
C4—C3—H3	126.6	C12—N1—C11	125.3 (5)
Fe1—C3—H3	126.6	C13—N1—C11	126.9 (5)
C5—C4—C3	108.4 (9)	C12—N2—C14	108.4 (5)
C5—C4—Fe1	70.0 (4)	C12—N2—C15	124.8 (6)
C3—C4—Fe1	69.0 (4)	C14—N2—C15	126.8 (6)
C5—C4—H4	125.8	C2—Fe1—C6	121.8 (3)
C3—C4—H4	125.8	C2—Fe1—C10	107.5 (3)
Fe1—C4—H4	125.8	C6—Fe1—C10	41.0 (2)
C4—C5—C1	108.7 (8)	C2—Fe1—C9	124.6 (3)
C4—C5—Fe1	70.2 (4)	C6—Fe1—C9	69.1 (2)
C1—C5—Fe1	69.5 (4)	C10—Fe1—C9	41.6 (2)
C4—C5—H5	125.7	C2—Fe1—C3	40.7 (4)
C1—C5—H5	125.7	C6—Fe1—C3	108.4 (3)
Fe1—C5—H5	125.7	C10—Fe1—C3	125.0 (3)
C10—C6—C7	108.6 (5)	C9—Fe1—C3	162.2 (4)
C10—C6—Fe1	69.5 (3)	C2—Fe1—C1	40.9 (4)
C7—C6—Fe1	70.2 (3)	C6—Fe1—C1	156.8 (3)
C10—C6—H6	125.7	C10—Fe1—C1	120.5 (3)
C7—C6—H6	125.7	C9—Fe1—C1	106.3 (3)
Fe1—C6—H6	125.7	C3—Fe1—C1	68.7 (3)
C6—C7—C8	107.5 (5)	C2—Fe1—C5	68.4 (4)
C6—C7—Fe1	68.6 (3)	C6—Fe1—C5	161.7 (3)
C8—C7—Fe1	69.7 (4)	C10—Fe1—C5	155.6 (3)
C6—C7—H7	126.3	C9—Fe1—C5	119.6 (3)
C8—C7—H7	126.3	C3—Fe1—C5	68.5 (3)
Fe1—C7—H7	126.3	C1—Fe1—C5	40.6 (4)
C9—C8—C7	108.0 (5)	C2—Fe1—C4	68.5 (4)
C9—C8—Fe1	69.0 (3)	C6—Fe1—C4	126.0 (3)
C7—C8—Fe1	69.3 (4)	C10—Fe1—C4	163.1 (3)
C9—C8—H8	126	C9—Fe1—C4	154.3 (4)
C7—C8—H8	126	C3—Fe1—C4	41.3 (4)
Fe1—C8—H8	126	C1—Fe1—C4	67.9 (4)
C8—C9—C10	108.8 (5)	C5—Fe1—C4	39.8 (3)
C8—C9—Fe1	70.7 (3)	C2—Fe1—C7	157.4 (4)
C10—C9—Fe1	69.0 (3)	C6—Fe1—C7	41.2 (2)
C8—C9—H9	125.6	C10—Fe1—C7	69.3 (2)
C10—C9—H9	125.6	C9—Fe1—C7	68.7 (2)
Fe1—C9—H9	125.6	C3—Fe1—C7	121.7 (4)
C6—C10—C9	107.1 (5)	C1—Fe1—C7	160.3 (3)
C6—C10—C11	127.6 (5)	C5—Fe1—C7	123.9 (3)
C9—C10—C11	125.2 (5)	C4—Fe1—C7	107.8 (3)
C6—C10—Fe1	69.5 (3)	C2—Fe1—C8	160.5 (4)
C9—C10—Fe1	69.4 (3)	C6—Fe1—C8	69.1 (2)
C11—C10—Fe1	125.5 (4)	C10—Fe1—C8	69.1 (2)
N1—C11—C10	111.0 (5)	C9—Fe1—C8	40.3 (2)
N1—C11—H11A	109.4	C3—Fe1—C8	156.7 (4)
C10—C11—H11A	109.4	C1—Fe1—C8	123.0 (3)
N1—C11—H11B	109.4	C5—Fe1—C8	106.3 (3)
C10—C11—H11B	109.4	C4—Fe1—C8	120.3 (4)
H11A—C11—H11B	108	C7—Fe1—C8	41.0 (3)
			
C5—C1—C2—C3	0.3 (8)	C8—C9—Fe1—C10	−120.0 (5)
Fe1—C1—C2—C3	59.8 (6)	C8—C9—Fe1—C3	−166.8 (10)
C5—C1—C2—Fe1	−59.5 (5)	C10—C9—Fe1—C3	−46.8 (11)
C1—C2—C3—C4	0.0 (9)	C8—C9—Fe1—C1	122.2 (4)
Fe1—C2—C3—C4	59.8 (6)	C10—C9—Fe1—C1	−117.8 (4)
C1—C2—C3—Fe1	−59.9 (5)	C8—C9—Fe1—C5	80.2 (5)
C2—C3—C4—C5	−0.3 (9)	C10—C9—Fe1—C5	−159.8 (4)
Fe1—C3—C4—C5	59.1 (6)	C8—C9—Fe1—C4	49.3 (9)
C2—C3—C4—Fe1	−59.4 (5)	C10—C9—Fe1—C4	169.3 (7)
C3—C4—C5—C1	0.5 (9)	C8—C9—Fe1—C7	−37.6 (4)
Fe1—C4—C5—C1	59.0 (5)	C10—C9—Fe1—C7	82.4 (4)
C3—C4—C5—Fe1	−58.5 (6)	C10—C9—Fe1—C8	120.0 (5)
C2—C1—C5—C4	−0.5 (9)	C4—C3—Fe1—C2	−118.1 (8)
Fe1—C1—C5—C4	−59.4 (6)	C2—C3—Fe1—C6	−117.8 (5)
C2—C1—C5—Fe1	58.9 (5)	C4—C3—Fe1—C6	124.2 (5)
C10—C6—C7—C8	0.0 (7)	C2—C3—Fe1—C10	−75.4 (6)
Fe1—C6—C7—C8	−59.1 (4)	C4—C3—Fe1—C10	166.6 (5)
C10—C6—C7—Fe1	59.1 (4)	C2—C3—Fe1—C9	−39.2 (13)
C6—C7—C8—C9	0.1 (7)	C4—C3—Fe1—C9	−157.2 (9)
Fe1—C7—C8—C9	−58.3 (4)	C2—C3—Fe1—C1	37.7 (6)
C6—C7—C8—Fe1	58.4 (4)	C4—C3—Fe1—C1	−80.4 (6)
C7—C8—C9—C10	−0.2 (7)	C2—C3—Fe1—C5	81.5 (6)
Fe1—C8—C9—C10	−58.6 (4)	C4—C3—Fe1—C5	−36.6 (5)
C7—C8—C9—Fe1	58.5 (4)	C2—C3—Fe1—C4	118.1 (8)
C7—C6—C10—C9	−0.1 (7)	C2—C3—Fe1—C7	−161.1 (5)
Fe1—C6—C10—C9	59.4 (4)	C4—C3—Fe1—C7	80.8 (6)
C7—C6—C10—C11	−179.1 (6)	C2—C3—Fe1—C8	162.7 (7)
Fe1—C6—C10—C11	−119.6 (6)	C4—C3—Fe1—C8	44.6 (10)
C7—C6—C10—Fe1	−59.5 (4)	C5—C1—Fe1—C2	118.9 (7)
C8—C9—C10—C6	0.2 (7)	C2—C1—Fe1—C6	49.5 (9)
Fe1—C9—C10—C6	−59.5 (4)	C5—C1—Fe1—C6	168.4 (6)
C8—C9—C10—C11	179.2 (5)	C2—C1—Fe1—C10	81.5 (6)
Fe1—C9—C10—C11	119.6 (6)	C5—C1—Fe1—C10	−159.6 (4)
C8—C9—C10—Fe1	59.7 (4)	C2—C1—Fe1—C9	124.4 (5)
C6—C10—C11—N1	−106.0 (7)	C5—C1—Fe1—C9	−116.7 (5)
C9—C10—C11—N1	75.1 (7)	C2—C1—Fe1—C3	−37.6 (6)
Fe1—C10—C11—N1	163.6 (4)	C5—C1—Fe1—C3	81.4 (6)
N1—C13—C14—N2	−1.3 (7)	C2—C1—Fe1—C5	−118.9 (7)
N2—C12—N1—C13	−0.4 (7)	C2—C1—Fe1—C4	−82.1 (6)
N2—C12—N1—C11	−179.5 (5)	C5—C1—Fe1—C4	36.8 (5)
C14—C13—N1—C12	1.1 (7)	C2—C1—Fe1—C7	−163.4 (8)
C14—C13—N1—C11	−179.9 (5)	C5—C1—Fe1—C7	−44.5 (10)
C10—C11—N1—C12	−86.7 (7)	C2—C1—Fe1—C8	165.2 (5)
C10—C11—N1—C13	94.4 (7)	C5—C1—Fe1—C8	−75.9 (5)
N1—C12—N2—C14	−0.4 (7)	C4—C5—Fe1—C2	81.9 (7)
N1—C12—N2—C15	−179.5 (5)	C1—C5—Fe1—C2	−38.0 (5)
C13—C14—N2—C12	1.1 (7)	C4—C5—Fe1—C6	−45.5 (13)
C13—C14—N2—C15	−179.8 (6)	C1—C5—Fe1—C6	−165.4 (8)
C3—C2—Fe1—C6	81.2 (6)	C4—C5—Fe1—C10	166.6 (7)
C1—C2—Fe1—C6	−159.3 (5)	C1—C5—Fe1—C10	46.7 (10)
C3—C2—Fe1—C10	123.8 (5)	C4—C5—Fe1—C9	−159.6 (6)
C1—C2—Fe1—C10	−116.7 (5)	C1—C5—Fe1—C9	80.5 (5)
C3—C2—Fe1—C9	166.5 (5)	C4—C5—Fe1—C3	37.9 (7)
C1—C2—Fe1—C9	−74.1 (6)	C1—C5—Fe1—C3	−82.0 (6)
C1—C2—Fe1—C3	119.5 (8)	C4—C5—Fe1—C1	119.9 (8)
C3—C2—Fe1—C1	−119.5 (8)	C1—C5—Fe1—C4	−119.9 (8)
C3—C2—Fe1—C5	−81.7 (6)	C4—C5—Fe1—C7	−76.7 (7)
C1—C2—Fe1—C5	37.8 (5)	C1—C5—Fe1—C7	163.4 (4)
C3—C2—Fe1—C4	−38.7 (6)	C4—C5—Fe1—C8	−118.0 (6)
C1—C2—Fe1—C4	80.7 (6)	C1—C5—Fe1—C8	122.1 (5)
C3—C2—Fe1—C7	45.9 (11)	C5—C4—Fe1—C2	−81.7 (6)
C1—C2—Fe1—C7	165.4 (7)	C3—C4—Fe1—C2	38.2 (6)
C3—C2—Fe1—C8	−159.4 (8)	C5—C4—Fe1—C6	163.9 (5)
C1—C2—Fe1—C8	−39.9 (12)	C3—C4—Fe1—C6	−76.2 (7)
C10—C6—Fe1—C2	79.9 (5)	C5—C4—Fe1—C10	−160.8 (10)
C7—C6—Fe1—C2	−160.3 (5)	C3—C4—Fe1—C10	−40.9 (15)
C7—C6—Fe1—C10	119.7 (5)	C5—C4—Fe1—C9	44.3 (11)
C10—C6—Fe1—C9	−38.6 (3)	C3—C4—Fe1—C9	164.2 (7)
C7—C6—Fe1—C9	81.1 (4)	C5—C4—Fe1—C3	−119.9 (9)
C10—C6—Fe1—C3	122.7 (5)	C5—C4—Fe1—C1	−37.5 (6)
C7—C6—Fe1—C3	−117.6 (5)	C3—C4—Fe1—C1	82.4 (6)
C10—C6—Fe1—C1	44.1 (8)	C3—C4—Fe1—C5	119.9 (9)
C7—C6—Fe1—C1	163.8 (7)	C5—C4—Fe1—C7	122.0 (6)
C10—C6—Fe1—C5	−160.5 (9)	C3—C4—Fe1—C7	−118.1 (6)
C7—C6—Fe1—C5	−40.7 (11)	C5—C4—Fe1—C8	78.9 (6)
C10—C6—Fe1—C4	165.2 (5)	C3—C4—Fe1—C8	−161.2 (5)
C7—C6—Fe1—C4	−75.1 (6)	C6—C7—Fe1—C2	48.2 (9)
C10—C6—Fe1—C7	−119.7 (5)	C8—C7—Fe1—C2	167.4 (8)
C10—C6—Fe1—C8	−81.9 (4)	C8—C7—Fe1—C6	119.2 (5)
C7—C6—Fe1—C8	37.8 (4)	C6—C7—Fe1—C10	−37.5 (4)
C6—C10—Fe1—C2	−118.7 (5)	C8—C7—Fe1—C10	81.7 (4)
C9—C10—Fe1—C2	122.8 (4)	C6—C7—Fe1—C9	−82.2 (4)
C11—C10—Fe1—C2	3.5 (6)	C8—C7—Fe1—C9	37.0 (3)
C9—C10—Fe1—C6	−118.5 (5)	C6—C7—Fe1—C3	81.7 (5)
C11—C10—Fe1—C6	122.2 (7)	C8—C7—Fe1—C3	−159.1 (4)
C6—C10—Fe1—C9	118.5 (5)	C6—C7—Fe1—C1	−161.0 (8)
C11—C10—Fe1—C9	−119.3 (6)	C8—C7—Fe1—C1	−41.8 (10)
C6—C10—Fe1—C3	−77.3 (5)	C6—C7—Fe1—C5	165.7 (4)
C9—C10—Fe1—C3	164.2 (5)	C8—C7—Fe1—C5	−75.1 (5)
C11—C10—Fe1—C3	45.0 (7)	C6—C7—Fe1—C4	124.8 (5)
C6—C10—Fe1—C1	−161.4 (4)	C8—C7—Fe1—C4	−116.0 (4)
C9—C10—Fe1—C1	80.1 (4)	C6—C7—Fe1—C8	−119.2 (5)
C11—C10—Fe1—C1	−39.2 (6)	C9—C8—Fe1—C2	−45.6 (10)
C6—C10—Fe1—C5	165.2 (7)	C7—C8—Fe1—C2	−165.5 (9)
C9—C10—Fe1—C5	46.7 (9)	C9—C8—Fe1—C6	82.0 (4)
C11—C10—Fe1—C5	−72.6 (9)	C7—C8—Fe1—C6	−38.0 (4)
C6—C10—Fe1—C4	−45.5 (13)	C9—C8—Fe1—C10	37.9 (3)
C9—C10—Fe1—C4	−163.9 (12)	C7—C8—Fe1—C10	−82.0 (4)
C11—C10—Fe1—C4	76.8 (13)	C7—C8—Fe1—C9	−119.9 (5)
C6—C10—Fe1—C7	37.7 (4)	C9—C8—Fe1—C3	169.9 (7)
C9—C10—Fe1—C7	−80.8 (4)	C7—C8—Fe1—C3	50.0 (9)
C11—C10—Fe1—C7	159.9 (6)	C9—C8—Fe1—C1	−75.7 (5)
C6—C10—Fe1—C8	81.7 (4)	C7—C8—Fe1—C1	164.4 (4)
C9—C10—Fe1—C8	−36.8 (3)	C9—C8—Fe1—C5	−116.8 (4)
C11—C10—Fe1—C8	−156.1 (6)	C7—C8—Fe1—C5	123.3 (4)
C8—C9—Fe1—C2	163.1 (4)	C9—C8—Fe1—C4	−157.7 (4)
C10—C9—Fe1—C2	−76.8 (5)	C7—C8—Fe1—C4	82.4 (5)
C8—C9—Fe1—C6	−81.9 (4)	C9—C8—Fe1—C7	119.9 (5)
C10—C9—Fe1—C6	38.1 (3)		
